# Recognition of industrial machine parts based on transfer learning with convolutional neural network

**DOI:** 10.1371/journal.pone.0245735

**Published:** 2021-01-28

**Authors:** Qiaoyang Li, Guiming Chen

**Affiliations:** Xi'an Research Institute of High-Tech, Xi’an, China; Vietnam National University, VIETNAM

## Abstract

As the industry gradually enters the stage of unmanned and intelligent, factories in the future need to realize intelligent monitoring and diagnosis and maintenance of parts and components. In order to achieve this goal, it is first necessary to accurately identify and classify the parts in the factory. However, the existing literature rarely studies the classification and identification of parts of the entire factory. Due to the lack of existing data samples, this paper studies the identification and classification of small samples of industrial machine parts. In order to solve this problem, this paper establishes a convolutional neural network model based on the InceptionNet-V3 pretrained model through migration learning. Through experimental design, the influence of data expansion, learning rate and optimizer algorithm on the model effectiveness is studied, and the optimal model was finally determined, and the test accuracy rate reaches 99.74%. By comparing with the accuracy of other classifiers, the experimental results prove that the convolutional neural network model based on transfer learning can effectively solve the problem of recognition and classification of industrial machine parts with small samples and the idea of transfer learning can also be further promoted.

## 1. Introduction

With the advancement of industrial technology and the transformation and development of modern factories, the manufacturing industry has gradually entered an unmanned and intelligent stage. In order to enable the entire production workshop to complete tasks systematically, orderly and independently, intelligent monitoring and maintenance are an important link. In order to improve the accuracy of monitoring and maintenance, accurate recognition of parts and machine parts of the entire industrial workshop is the primary goal.

In order to realize unmanned and intelligent factories, in literature [1], a method of recognizing the position, location and orientation of irregular machine parts with a complex outline of the external contour is suggested. In literature [2], a geometric part measurement system for shaft parts based on machine vision is presented. These studies have potential applications for the development of manufacturing. However, there is no research on the identification of small samples of parts in the whole factory.

In order to realize the accurate recognition of various machine parts, it is necessary to research the image recognition of industrial machine parts. Image recognition refers to the technology of recognition of images with the same characteristics in different modes and environments via the use of computers to process, analyze and understand a large number of images. Traditional image recognition processes include image acquisition, image preprocessing, feature extraction and image recognition. Literature [3] evaluates the performance of the developed MLP and SOM NN based classifier for detection of four conditions of three phase induction motor and examined the results. The cross-validation method in the paper is worth learning, but its calculation is more complicated and there are fewer classification types, and the effect cannot be determined in the face of small samples and multiple classifications, and the accuracy of the results can be further improved. At the same time, the literature only studies the induction motor, and does not extend to the parts of the entire factory. In recent years, deep learning based on convolutional neural networks (CNN) [4] have been used for various types of image recognition and achieved very good results. In 2012, the CNN network model reduced the error rate from 25.77% to 15.319% for the first time in the ImageNet Large Acale Visual Recognition Challenge (ILSVRC) competition. And at the 2017 competition, the lowest error rate has dropped to 2.251%.

Although CNN plays a certain role in image feature extraction and recognition, they all require a large amount of sample data for iterative training of neural networks. However, for this specific field of industrial machine component recognition, there is not enough sample data. If the small sample data is directly trained by CNN, the obtained model will have a large error and is difficult be promoted. In order to solve the problem of image recognition with small sample, Gene Kitamura realized the detection and recognition of small samples of ankle fractures through a new training and multi-view merged CNN [5] and the final recognition accuracy reached 81%. Jufeng Yang proposed a self-paced learning algorithm for small sample recognition of clinical rare skin diseases [6]. Qian Huang proposes a new blood cell classification framework based on medical hyperspectral imaging in order to complete the task of white blood cell classification under small sample training, combining the modulated Gabor wavelet and CNN kernel [7]. In the field of industrial machine parts recognition, there are few algorithms related to its specific research. In order to solve the problem of recognition and classification of industrial machine parts under the condition of small samples, a recognition method of industrial machine parts based on transfer learning [8–15] with CNN is proposed. Not only can further improve the recognition accuracy, but also save the time of model training. And the related parameters are flexible and can be adjusted according to different target images and recognition tasks. It also has a good promotion effect in related research and industrial practice.

The first part of this article explains the necessity and current situation of the research on this subject, aiming to solve the problem that the factory will realize the accurate classification and recognition of industrial machine parts in the plant under a small sample data set in the future. The second part introduces and constructs the basic theory and basic framework of transfer learning based on CNN. The third part introduces the source and classification of the experimental data set, designs the experimental grouping and studies the influence of different variables on the model training effect. The fourth part summarizes the experiment and puts forward the research direction of the next stage.

## 2. Construction of convolutional neural network model based on transfer learning

### 2.1. Convolutional neural network

CNN in deep learning is a neural network specifically used to process data with a grid structure. CNN includes multiple convolutional layers, pooling layers, and fully connected layers [16]. Based on the feed-forward neural network, the model updates the parameter weights by iteratively training the loss function to feed back the errors to each network layer. As the iterative training progresses, the parameter weights are continuously updated to achieve the desired training effect.

The role of the convolution layer is to perform feature mapping of the input through the convolution kernel to extract the features of the image [17–19]. The convolution operation formula is

$$S(i,j)=(K*I)(i,j)=\sum_{m}\sum_{n}I(i+m,j+n)K(m,n)$$ (1)

Where ***S*** (*i*, *j*) represents the output tensor of the convolution layer, ***I*** (*i+m*, *j+n*) represents the input tensor of the convolution layer, ***K*** (*m*, *n*) represents the convolution kernel, *i*, *j* represent the coordinate values of the tensor, *m*, *n* represent the coordinate value of the convolution kernel.

The function of the pooling layer is to further process the feature mapping results obtained by the convolution operation. The pooling function will statistically summarize the feature values of a position in the plane and its adjacent positions, and use the summarized result as the value of this position in this plane. Common pooling functions include average pooling, maximum pooling, and random pooling. Taking the maximum pooling function with a size of 2 × 2 as an example, its calculation formula is

$$f_{pool}=\mathrm{Max}(s_{i,j},s_{i+1,j},s_{i,j+1},s_{i+1,j+1})$$ (2)

Where *f*_*pool*_ represents the result after pooling, *s*_*i*,*j*_ represents the element whose position on the feature map tensor is (*i*, *j*).

The fully connected layer is a dimensionality reduction and tiling of the results obtained by the convolutional layer and the pooling layer and then performs non-linear transformation through the activation function. Finally, the results are input into the classifier for classification.

### 2.2. Transfer learning and model building

#### 2.2.1. Transfer learning

Transfer learning is a new machine learning method that uses existing knowledge to solve different but related domain problems. For CNN, the convolutional layer and pooling layer are retained. The CNN's convolutional layer trained on a large amount of sample data can perform feature extraction on another image data. The extracted feature vector is processed by the pooling layer and then processed. Add a new fully connected layer to form a new network model. To put it simply, it retains the model's feature extraction and recognition capabilities and adds new object orientation to enable itself to complete new image recognition and classification tasks.

#### 2.2.2. InceptionNet-V3 convolutional network model

**Fig 1:**
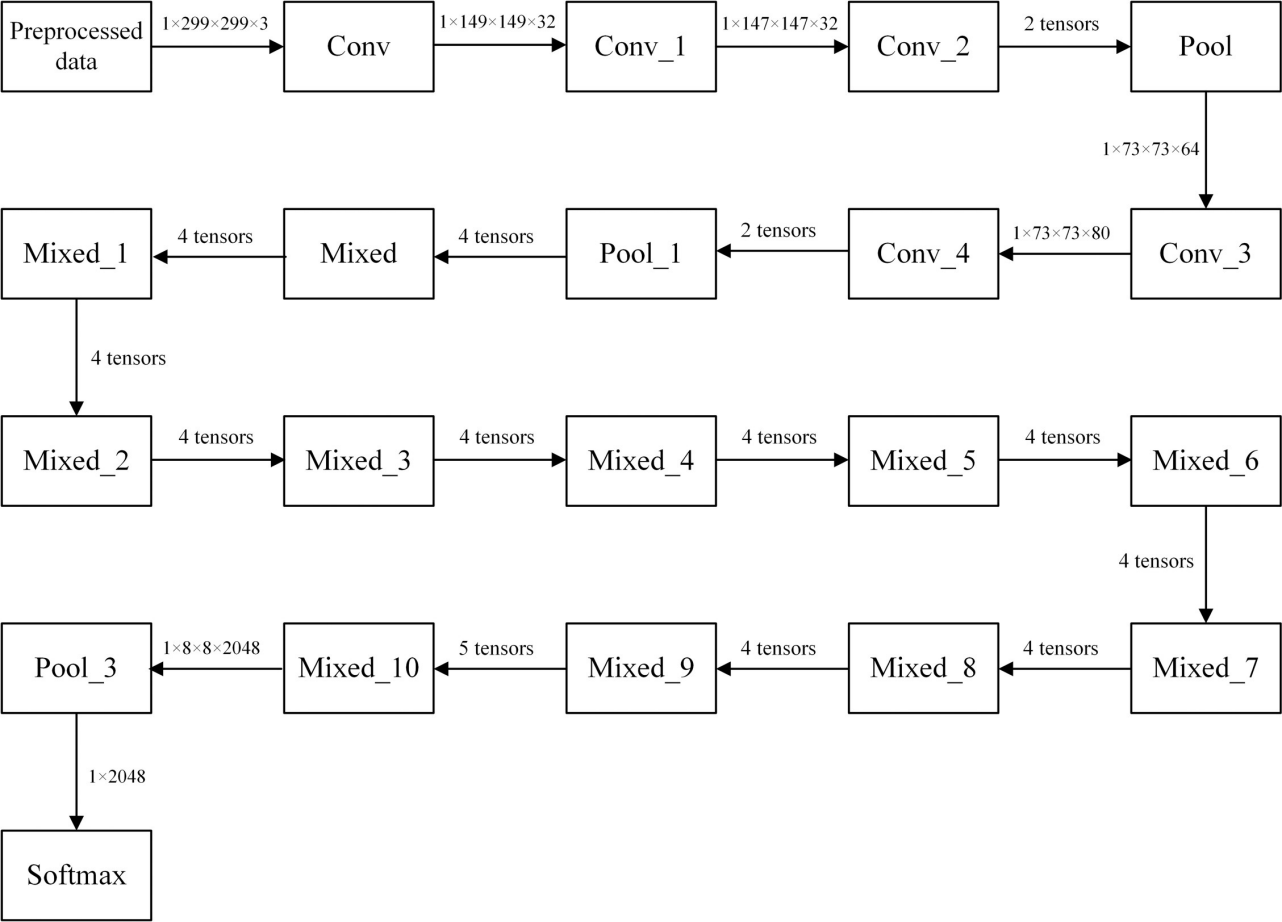
InceptionNet-V3 model architecture diagram.

Commonly used pretrained models contain Resnet [20], VGG [21], Alexnet [22, 23] and InceptionNet-V3 [24], etc. Compared with other models, InceptionNet-V3's classifier has a smaller number of operations, which can reduce the training time, and can also reduce the structural redundancy through convolution. At the same time, we can see from the literature [25] that on the classification problem based on transfer learning, InceptionNet-V3 achieved good results. Therefore, this article first considers the use of InceptionNet-V3 for transfer learning. This paper uses InceptionNet-V3 convolutional network model for transfer learning. InceptionNet-V3 was proposed in the paper "Rethinking the Inception Architecture for Computer Vision" in December 2015. InceptionNet-V3 has two main improvements over InceptionNet-V2. The first is to optimize the structure of the Inception Module. The second is to introduce a larger two-dimensional convolution into two smaller one-dimensional convolutions in InceptionNet-V3. This is called the "Factorization into small convolutions" idea. This asymmetric convolutional structure split is more effective than symmetric structures in dealing with more and richer spatial features and increasing feature diversity. The architecture diagram of the InceptionNet-V3 model is shown in Fig 1.

The InceptionNet-V3 model has a total of 46 layers and consists of 11 Inception modules, including 96 convolutional layers. The convolutional layers are implemented by TensorFlow's Slim tool.

#### 2.2.3. Construction of transfer learning model

**Fig 2:**
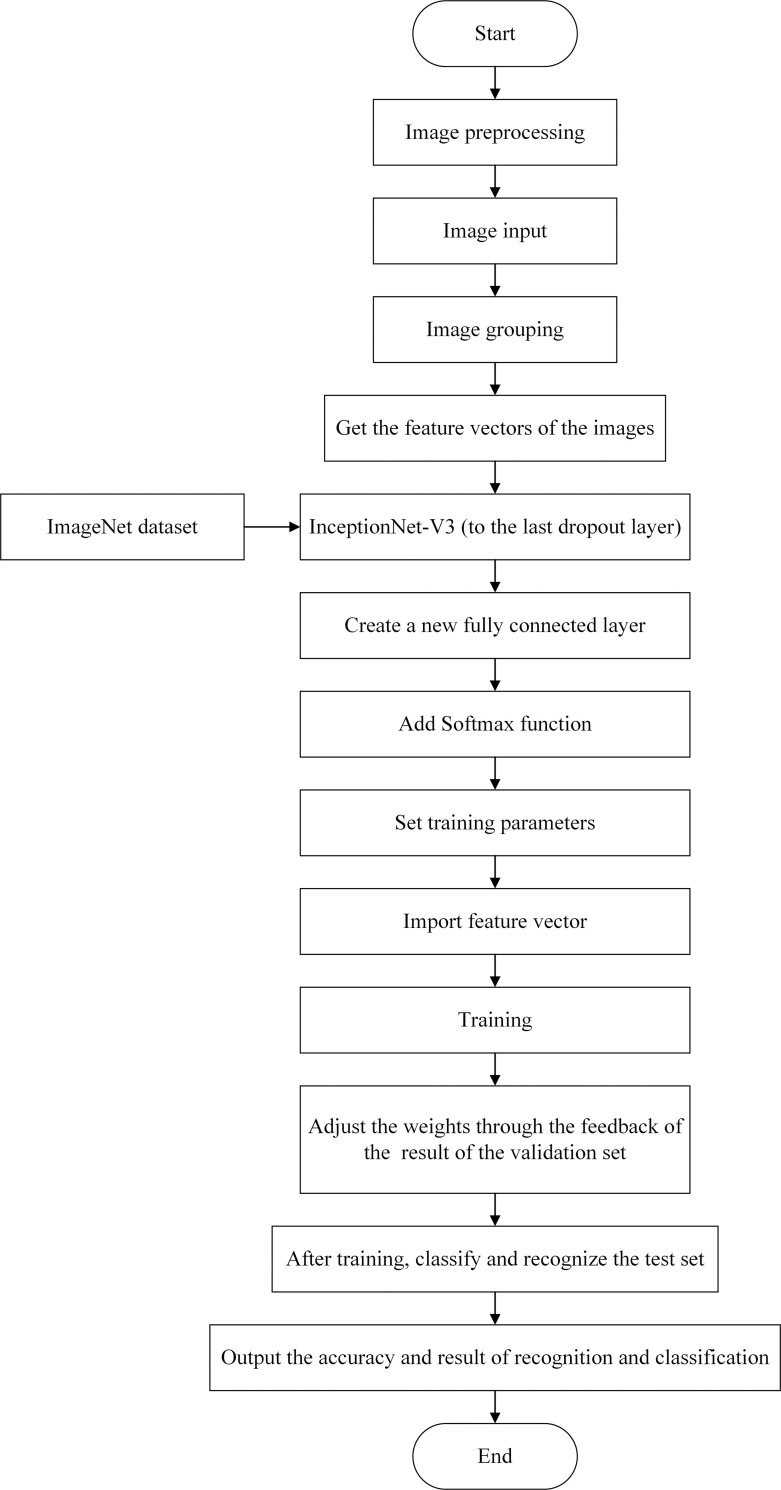
Modified flowchart of recognition of industrial machine parts.

InceptionNet-V3 completed training on the ImageNet data set and the number of training samples reached 1.2 million [26–28]. However, the number of images of industrial machine parts is not yet large enough. Therefore, the transfer learning method is used to recognize and classify industrial machine parts based on the InceptionNet-V3 model. For the trained InceptionNet-V3 model, the parameters of all convolutional layers are retained and the last fully connected layer is replaced. The previous network layer of this fully connected layer is called the bottleneck layer, which is the last Dropout layer in InceptionNet-V3. The results of the new fully connected layer are passed to a Softmax layer, and new recognition tasks can be processed. The modified module process of industrial machine parts recognition is shown in Fig 2.

The gradient descent optimizers that can be used in training mainly include stochastic gradient descent, AdaGrad, RMSProp and Adam optimizers. Take the Adam algorithm as an example to introduce the principle of its optimization method.

First set the global learning rate *σ*. The exponential decay rate of moment estimation is *ρ*_1_ and *ρ*_2_, and in the interval [0,1], the default is 0.9 and 0.990. The initialized parameter is *ω*. A small constant created for numerical stability *δ*, default takes *δ* = 10^−8^. The first and second moment variables *s* and *r* with initial values of 0. And an event step count *t*, *t* is initialized with *t* = 0. Then execute the following steps in a loop without stopping before the stop condition.

(1) Take out the mini-batch data of *m* samples from the training set {*x*_1_,*x*_2_,⋯,*x*_*m*_}, and the target corresponding to the data is represented by *y*_*i*_.

(2) Calculate the gradient as follows.

$$g\leftarrow \frac{1}{m}\nabla_{m}\sum_{i}L(f(x_{i};\omega ),y_{i})$$ (3)

(3) The refresh time steps are as follows.

t←t+1 (4)

(4) Update the first-order partial moment estimation.

$$s\leftarrow \rho_{1}r+(1-\rho_{1})g⊙g$$ (5)

(5) Update the second-order biased moment estimation。

$$r\leftarrow \rho_{2}r+(1-\rho_{2})g⊙g$$ (6)

(6) Correct the deviation of the first-order moment.

$$\hat{s}\leftarrow \frac{s}{1-\rho_{1}^{t}}$$ (7)

(7) Correct the deviation of the second moment.

$$\hat{r}\leftarrow \frac{r}{1-\rho_{2}^{t}}$$ (8)

(8) Calculate the update amount of the parameter.

$$\Delta \omega =-\sigma \frac{\hat{s}}{\sqrt{\delta +\hat{r}}}$$ (9)

(9) Update the parameters according to Δ*ω*.

ω=ω+Δω (10)

Assume that the output of the original neural network is *y*_1_, *y*_2_, … *y*_n_, then the output after softmax [29] regression processing is

$$\mathrm{softmax}(y)=\frac{\exp(y_{i})}{\sum_{i=1}^{n}\exp(y_{i})}$$ (11)

## 3. Model training and analysis of experimental results

### 3.1. Data set

**Fig 3:**
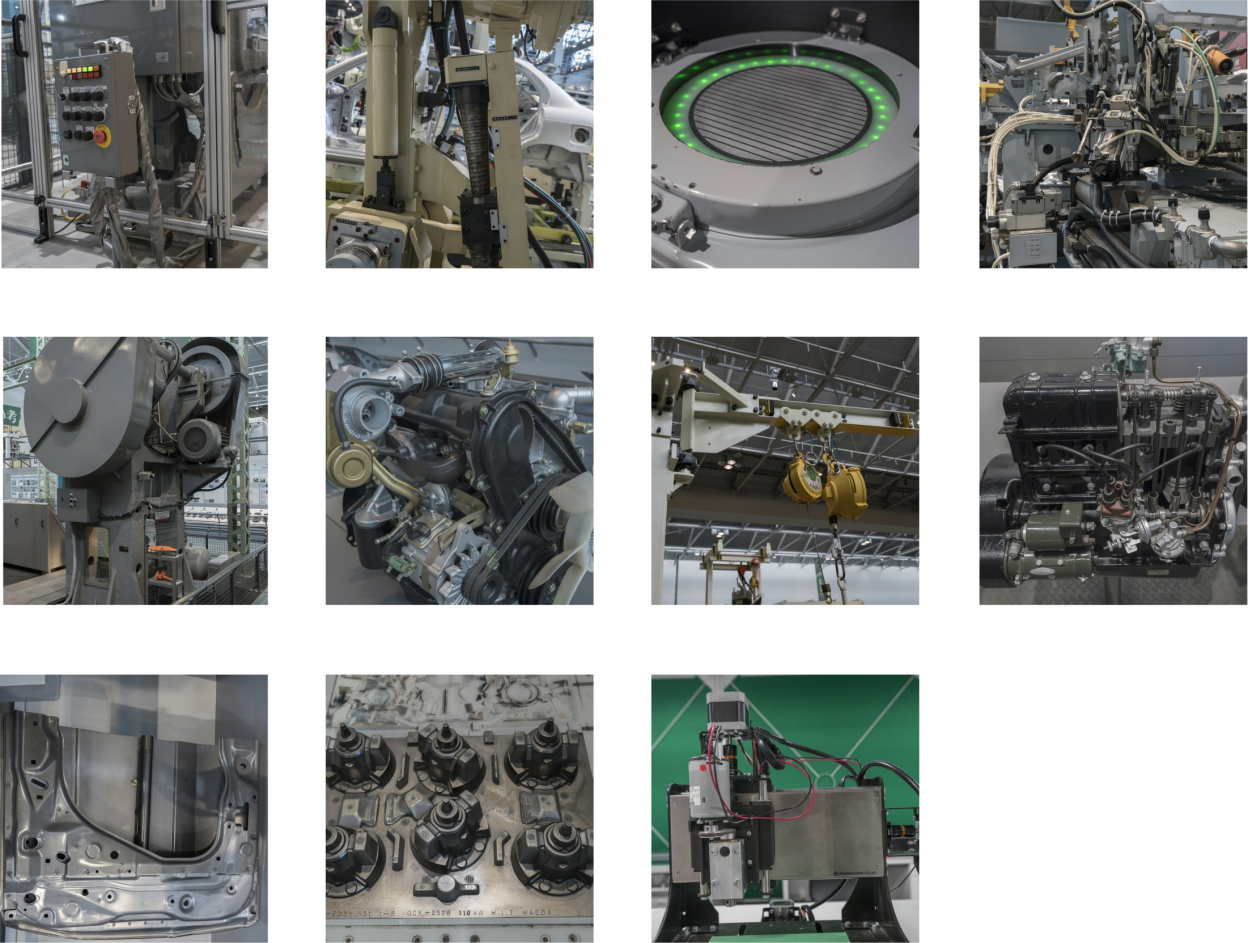
Examples of dataset image.

The data set used in the experiment came from a field shooting of a factory, includeing 11 types of industrial machine parts such as control panels, plate, robotic arms, and assembly, etc. with a total of 1002 images. As shown in Fig 3A–3K are sample images of each type of image set. Table 1 summarizes the number of each category. In the experiment, the image is augmented to 4008 images through rotation, flip, etc. 80% of the data set is used for training, 10% is used for validation and 10% is used for testing.

**Table 1:** Summary of the number of each category.

Numbers	Types of industrial machine parts	Number of dataset
1	Control panels	56
2	Robotic arms	79
3	Interactive module	32
4	Assembly	79
5	Big machines	146
6	Engine	137
7	Hangar	52
8	Old Machinery	72
9	Plates	81
10	Tech parts	193
11	Others	75

In Fig 3, from top to bottom, from left to right, control panels, robotic arms, interactive module, assembly, big machines, engine, hangar, old machinery, plates, tech parts and others are in turn.

**Fig 4:**
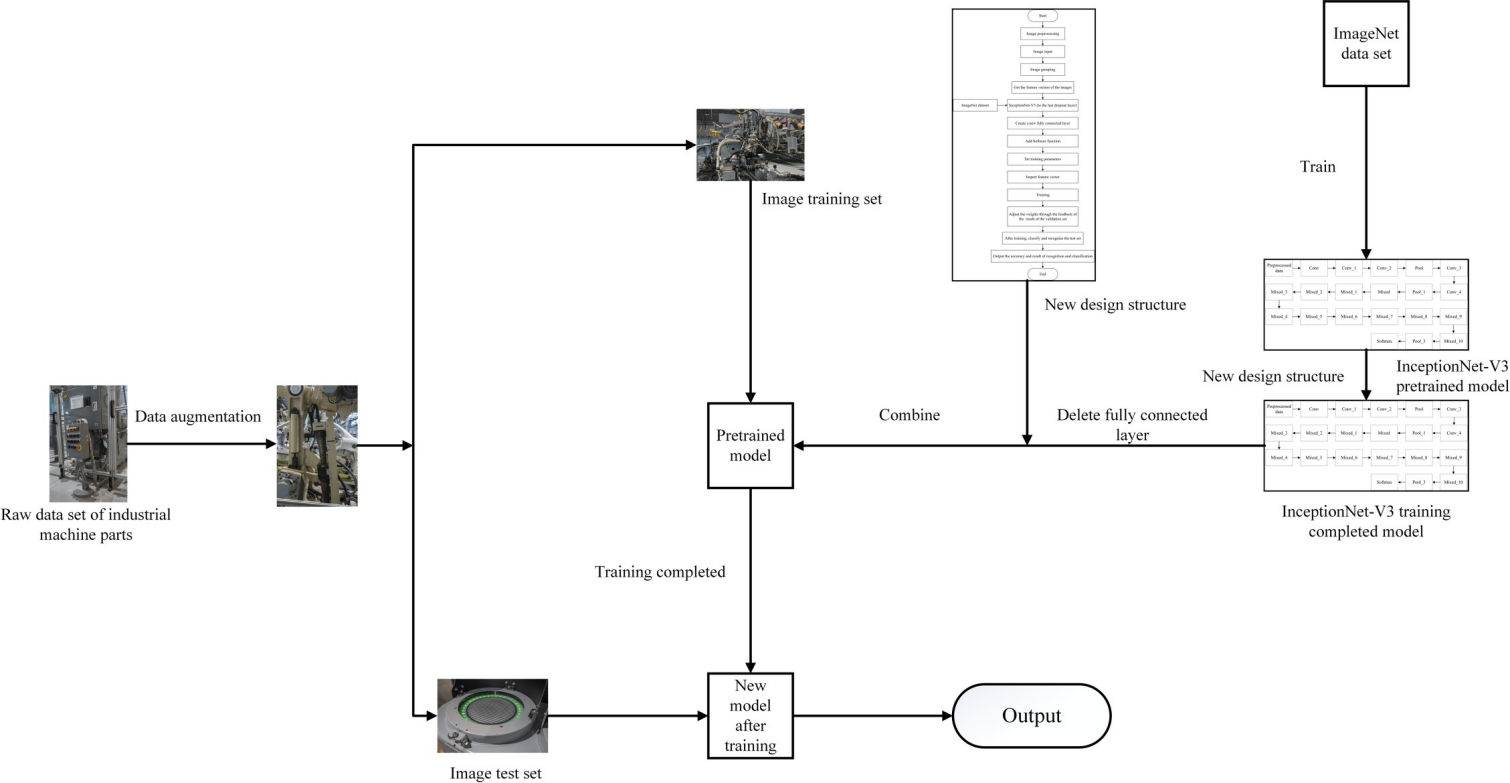
Classification and recognition process of industrial machine parts based on transfer learning.

The classification and recognition process of industrial machine parts based on transfer learning can be obtained as shown in Fig 4.

Compared with other models, the novelty of the model in the paper is as follows:

1. The training process is simplified, the amount of calculation is reduced, and training time is saved.
2. In the case of limited samples, better training results can be achieved.
3. The redundancy of the structure is reduced, which is conducive to further expansion, improvement and supplementation.

### 3.2 Experimental design

The experiments were completed under the software environment of Python 3.7.0 and TensorFlow 1.15.0. In the hardware environment, the CPU uses Intel Corei5-6200U and the main frequency is 2.3GHz; the GPU uses NVIDIA GeForce 950M and 2GB video memory.

The hyperparameters of the training neural network are set as follows: the initial learning rate is set to 0.01, the batch size is set to 32 and the total number of iteration training times is set to 40,000.

In order to get better training results, the experiment set different contrast experimental groups:

1. Comparison of the original data set (1002 images) and the model of the data set (4008 images) after simple flipping, folding and other operations.
2. Comparison of models obtained under different learning rates.
3. Comparison of models obtained using different gradient descent optimizers.

### 3.3 Analysis of experimental results

#### 3.3.1 Impact of image data augmentation on models

**Fig 5:**
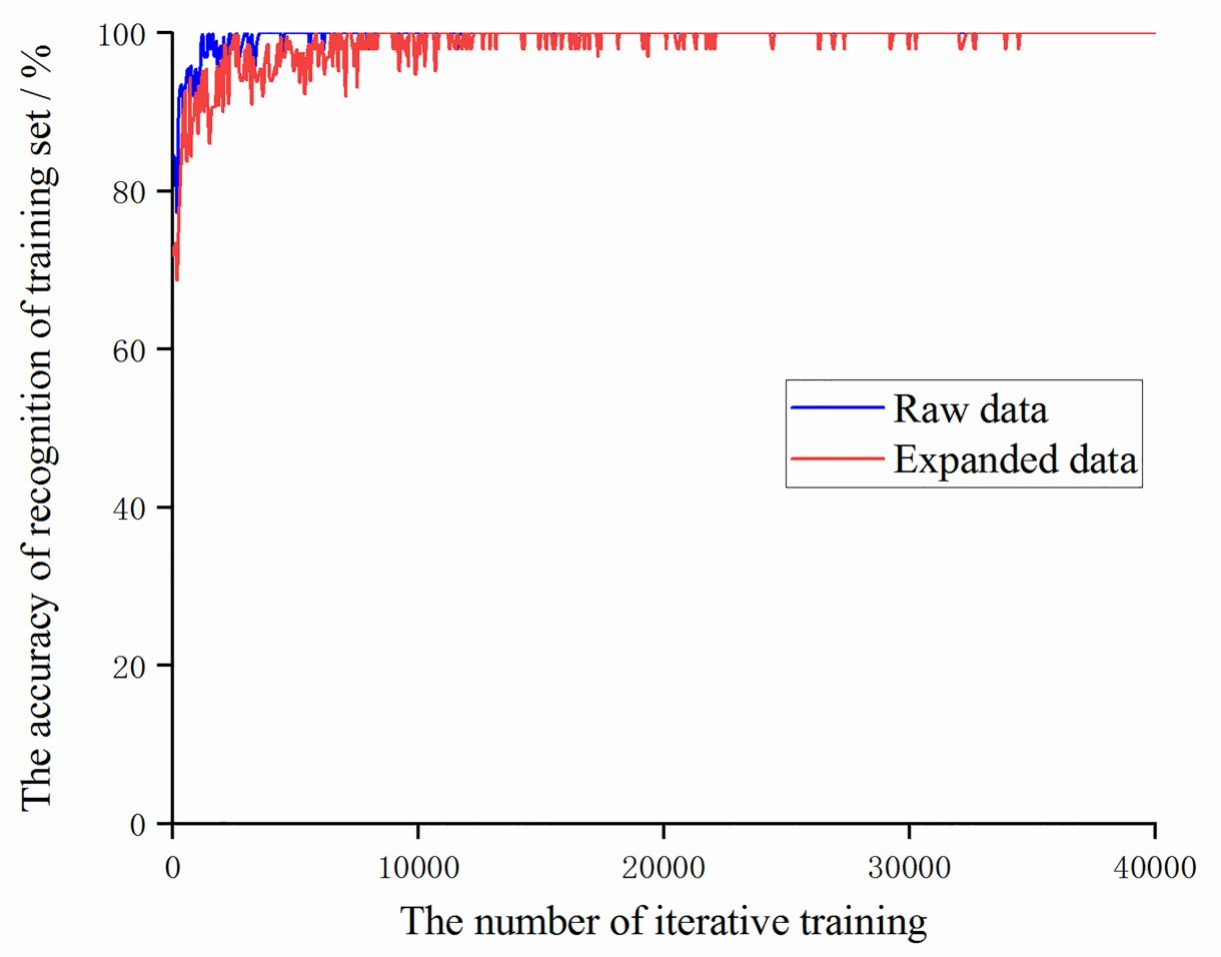
The trend of the accuracy of the training set of the original data and the augmented data with the number of iterative training.

**Fig 6:**
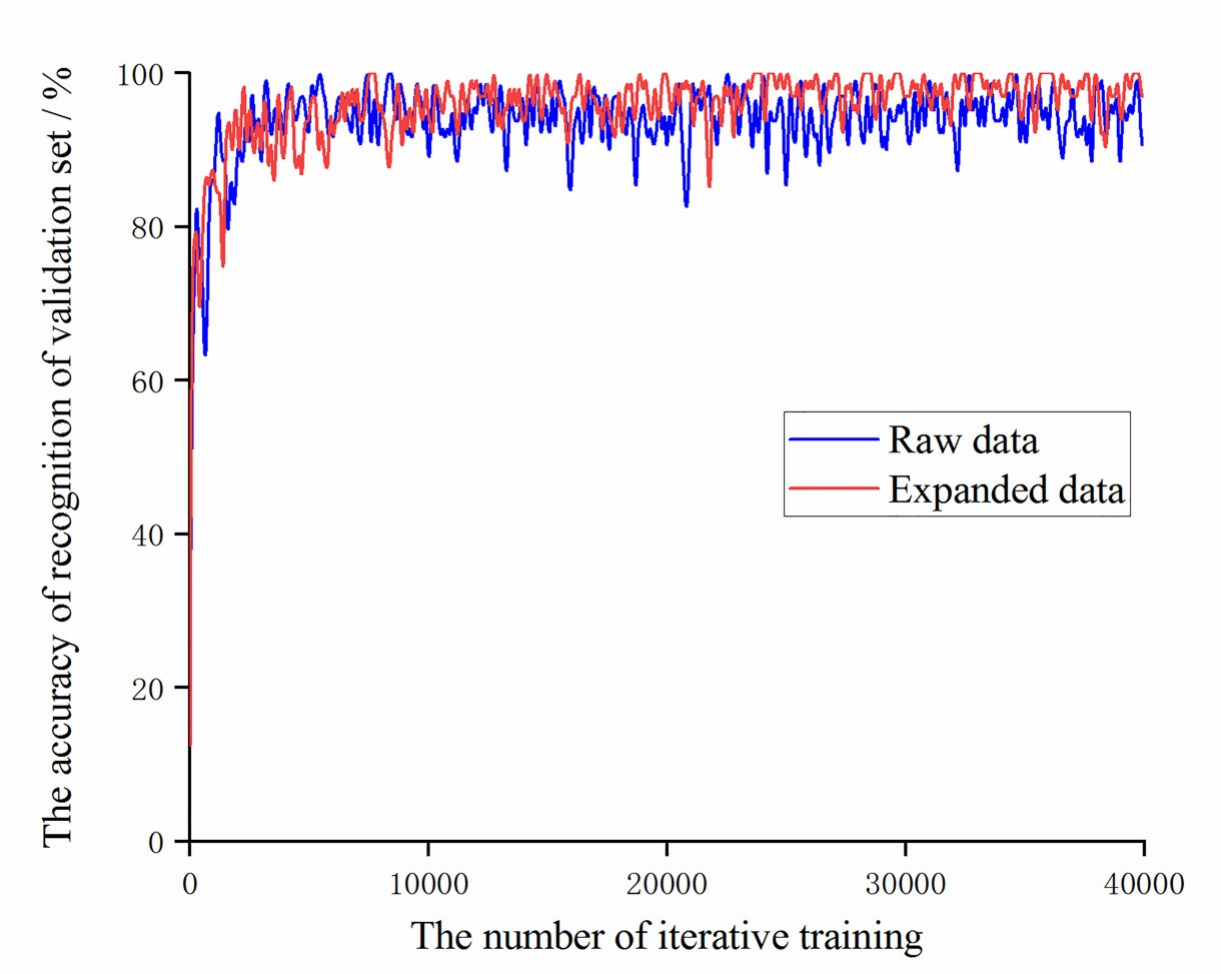
The trend of the accuracy of the validation set of the original data and the augmented data with the number of iterative training.

For this experimental sample, under the condition that the learning rate is set to 0.01 and the optimizer uses a stochastic gradient descent optimizer, the two trained models are compared. The trend of the accuracy of the training set and validation set with the number of iterative trainings is shown in the Figs 5 and 6.

**Fig 7:**
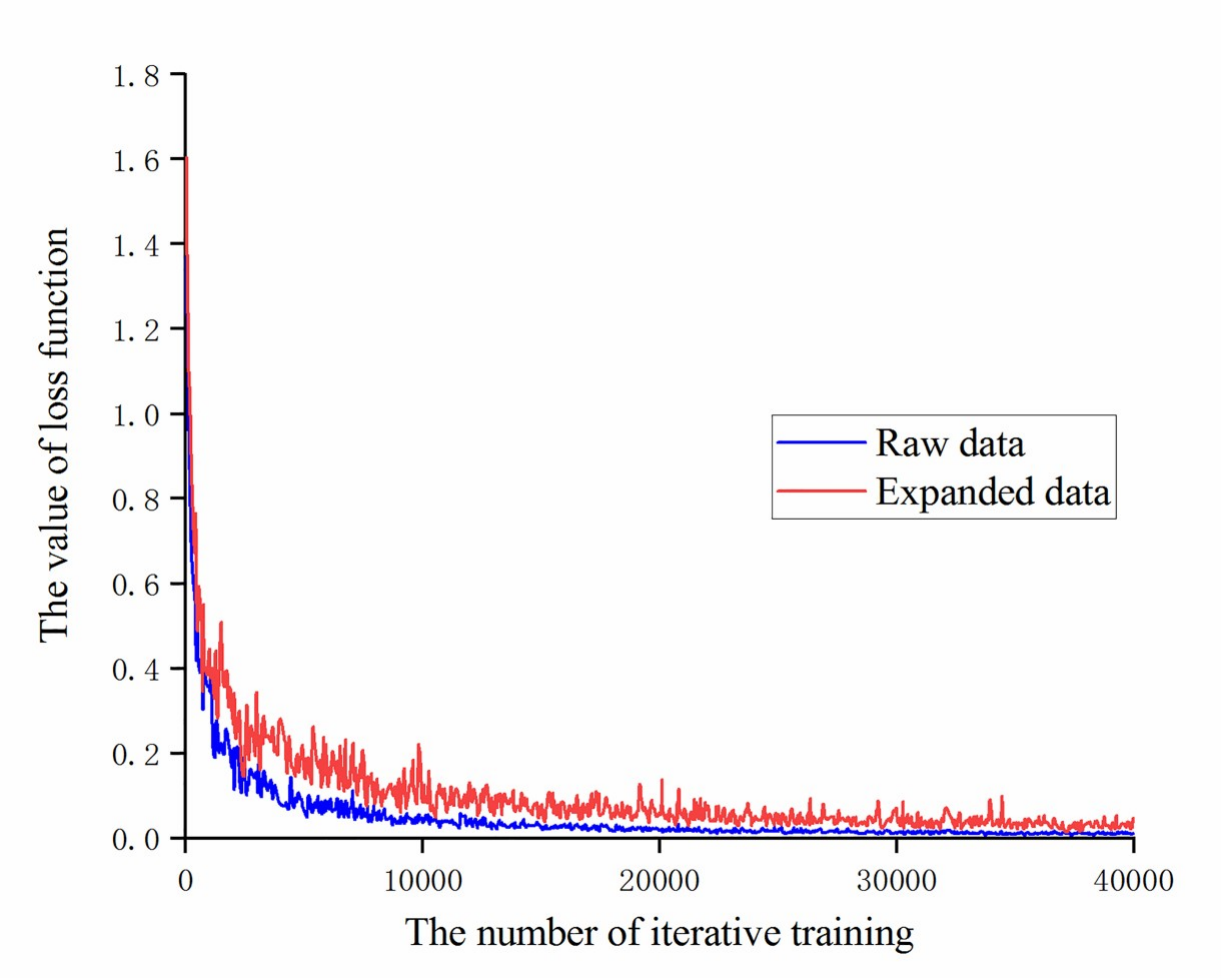
The change trend of the loss function value of the original data and the augmented data with the number of iterative training.

It can be seen that during the training process, the training set accuracy of the original data and the augmented data both reached 100% after 10,000 iterations of training. The accuracy of recognition of the augmented data validation set is significantly higher than the original data after 25,000 iterations of training. Then compare the value of the loss function during training, as shown in Fig 7. It can be found that the value of the loss function of the augmented data is always slightly higher than the value of the loss function of the original data, which also shows that as the number of data sets increases, it is necessary to increase the number of trainings to obtain more ideal training results.

After the model training is completed, the test set divided by the experiment is used to test the model and the training effect of training data set and validation data set is summarized as shown in Table 2.

**Table 2:** Comparison of model accuracy between original and augmented data.

Different data sets	Training set accuracy / %	Validation set accuracy / %	Test set accuracy / %
Raw data	100.00	90.62	96.40
Augmented data	100.00	96.88	97.92

From Table 2, after 40,000 iterations of training, the accuracy of recognition of the training set has reached 100%. By expanding the image through operations such as rotation and folding, the accuracy of recognition of the validation set of the model is increased by 6.26 percentage points, and the accuracy of recognition of the test set is increased by 1.52 percentage points. The accuracy of recognition of the test set is improved, but the amplitude is not large. The reason is that operations such as rotation and folding do not change the features and quality of the image. At the same time, the model has been trained on large data sets due to transfer learning model. A better feature extraction ability is obtained, so on the other hand, the effect of simple expansion of small sample data is also weakened.

#### 3.3.2 Impact of different learning rates on models

**Fig 8:**
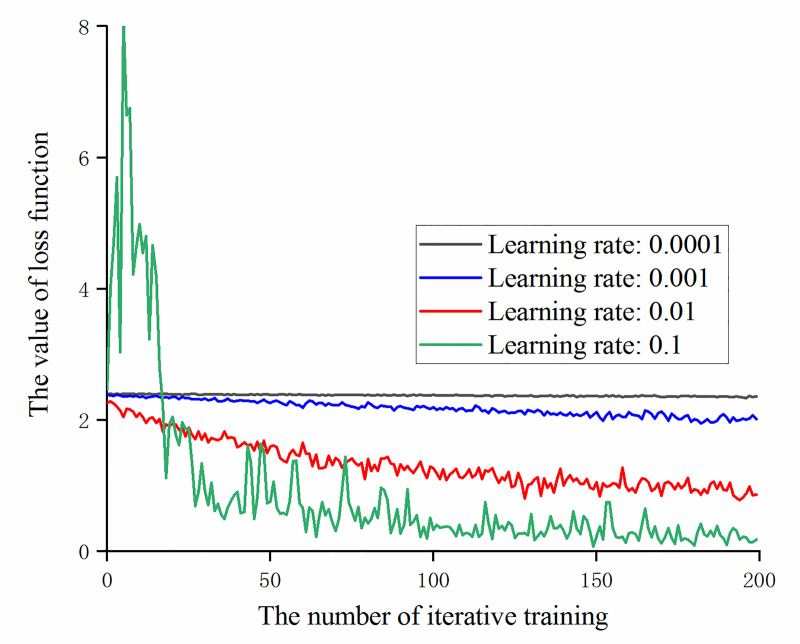
The trend of the loss function value at different learning rates with the number of iterative training.

For the augmented data, under the condition of using a stochastic gradient descent optimizer, different learning rates are set and the loss function value is observed during the training of the model. Compare the loss function values of the first 200 iterations of training with learning rates [30–32] of 0.0001, 0.001, 0.01 and 0.1 respectively, as shown in Fig 8.

**Fig 9:**
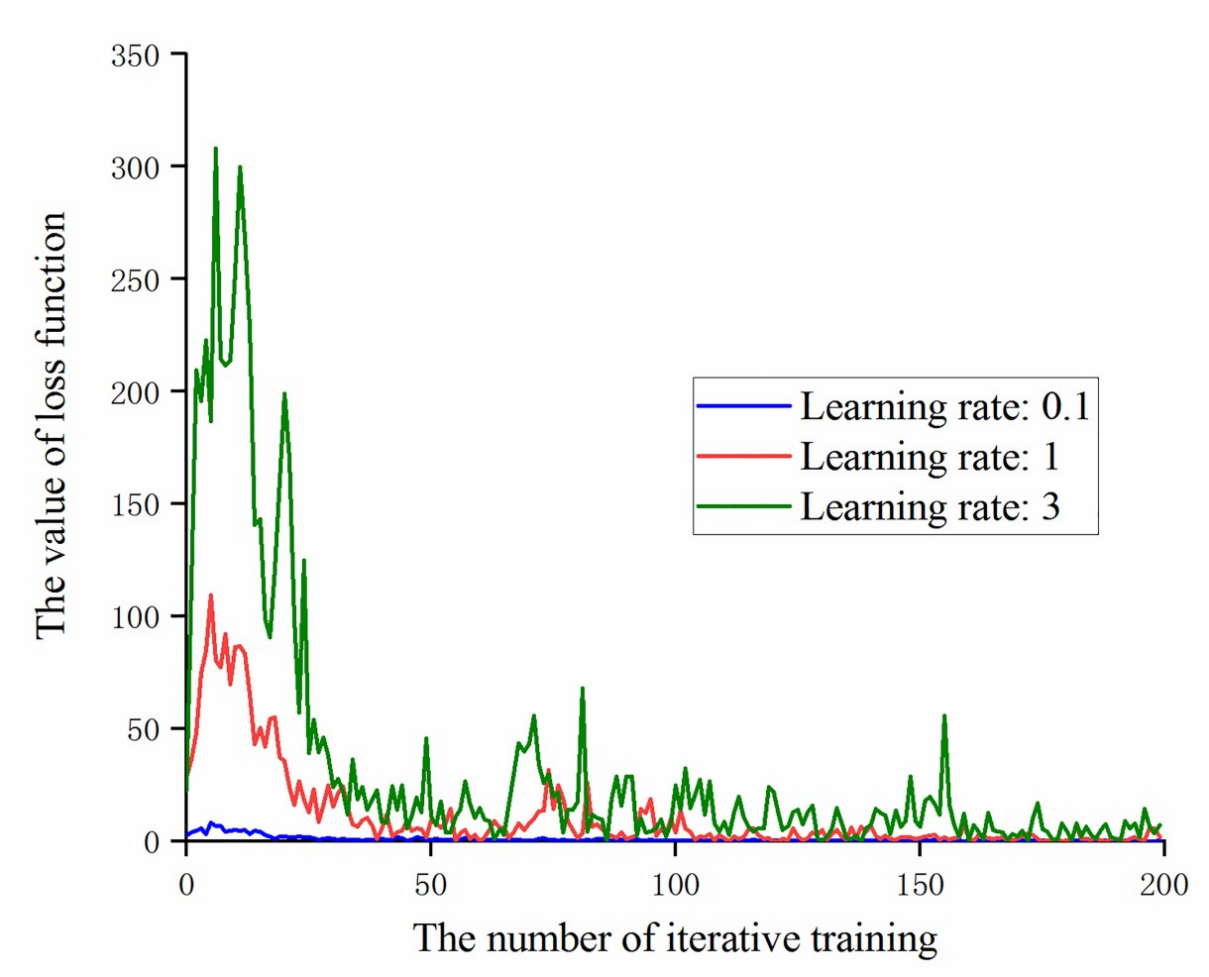
The trend of loss function value with the number of iterative training at large learning rates.

It can be obtained from Fig 8 that if the learning rate is too small (for example, the learning rate is set to 0.0001), the value of the loss function will fluctuate continuously, but the convergence cannot be reduced. The reason is that the learning rate is too small, the convergence speed is slow and no obvious convergence effect can be obtained with a small number of iterative training times. At the same time, it can be found that in the case of the experimental samples and settings, when the learning rate is 0.1, a significant gradient explosion occurs at the beginning of training. In order to eliminate the chance, further testing whether the learning rate is too large will cause a gradient explosion. Under the same conditions, the learning rate is set to 1 and 3 for iterative training. The loss function value of the first 200 iterations is shown in Fig 9.

It can be obtained from Fig 9 that with the continuous increase of the learning rate, the peak value of the loss function at the beginning of training also increases and the effect of the gradient explosion is more significant. At the same time, in the subsequent iterative training, the value of the loss function continuously oscillated, proving that the model parameters are updated too quickly and the difference is large, destroying the previously trained weight information, causing the model to fail and the transfer learning to be meaningless.

**Fig 10:**
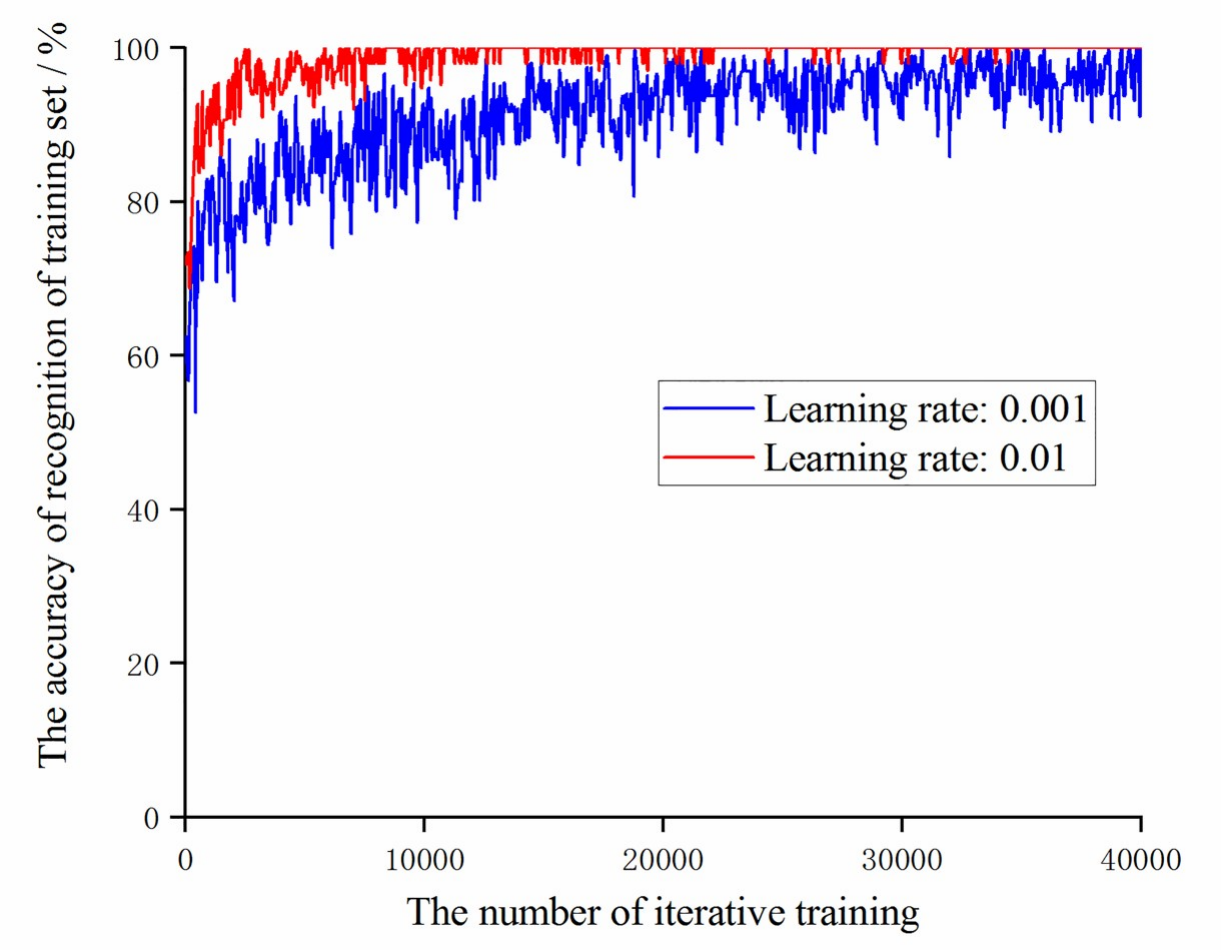
The trend of the accuracy of the training set with the number of iterative training at learning rates of 0.001 and 0.01.

**Fig 11:**
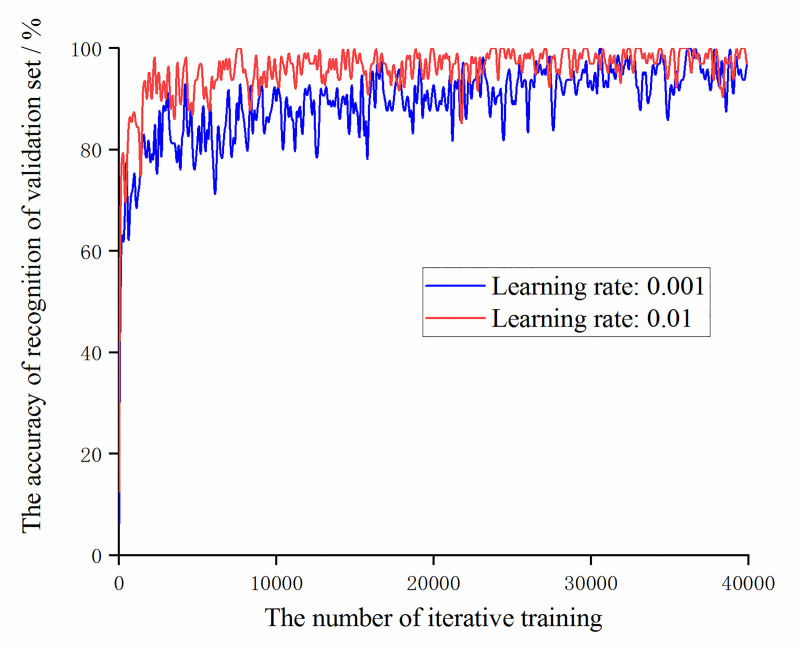
The trend of the accuracy of the validation set with the number of iterative training at learning rates of 0.001 and 0.01.

After filtering the learning rate, select the accuracy of the data training set and validation set of the models at learning rates of 0.001 and 0.01, as shown in Figs 10 and 11.

**Fig 12:**
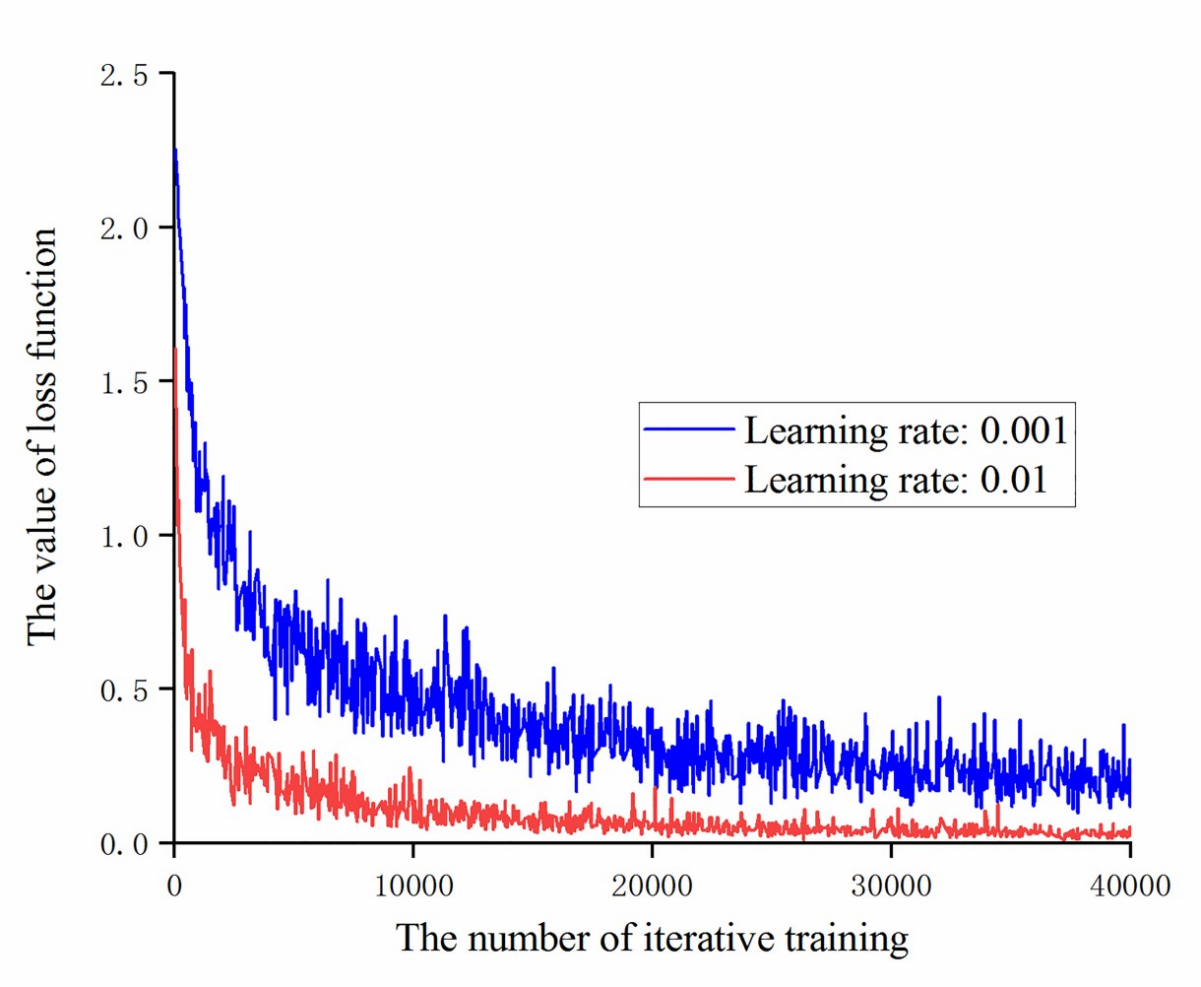
The trend of loss function value with the number of iterative training at learning rates of 0.001 and 0.01.

It can be obtained from Figs 10 and 11, during the iterative training process, the accuracy of recognition of the training set and validation set of the model with a learning rate of 0.01 is always higher than the model with a learning rate of 0.001. Then compare the change of the loss function value with the number of iterative training times under the two learning rate values, as shown in Fig 12.

It can be obtained from Fig 12 that under the experimental samples, neither of the two models of learning rate take the case of non-convergence or gradient explosion. The loss function value of the model with a learning rate of 0.01 is smaller than that of the model with a learning rate of 0.001. The convergence is faster and the fluctuation range is smaller.

After 40,000 iterations of training are completed, the test set is used to test the two learning rate models and the training effect of training data set and validation data set is summarized as shown in Table 3.

**Table 3:** Comparison of model accuracy rates at different learning rates.

Different learning rates	Training set accuracy / %	Validation set accuracy / %	Test set accuracy / %
0.001	100.00	96.88	91.15
0.01	100.00	96.88	97.92

According to Table 3, after 40,000 iterations of training, the accuracy of recognition of the validation set of the model with a learning rate of 0.01 is the same as that of the model with a learning rate of 0.001, but the accuracy of recognition of the test set of the model with a learning rate of 0.01 is increased by 6.77 percentage points.

#### 3.3.3 Impact of different gradient descent optimizers on models

**Fig 13:**
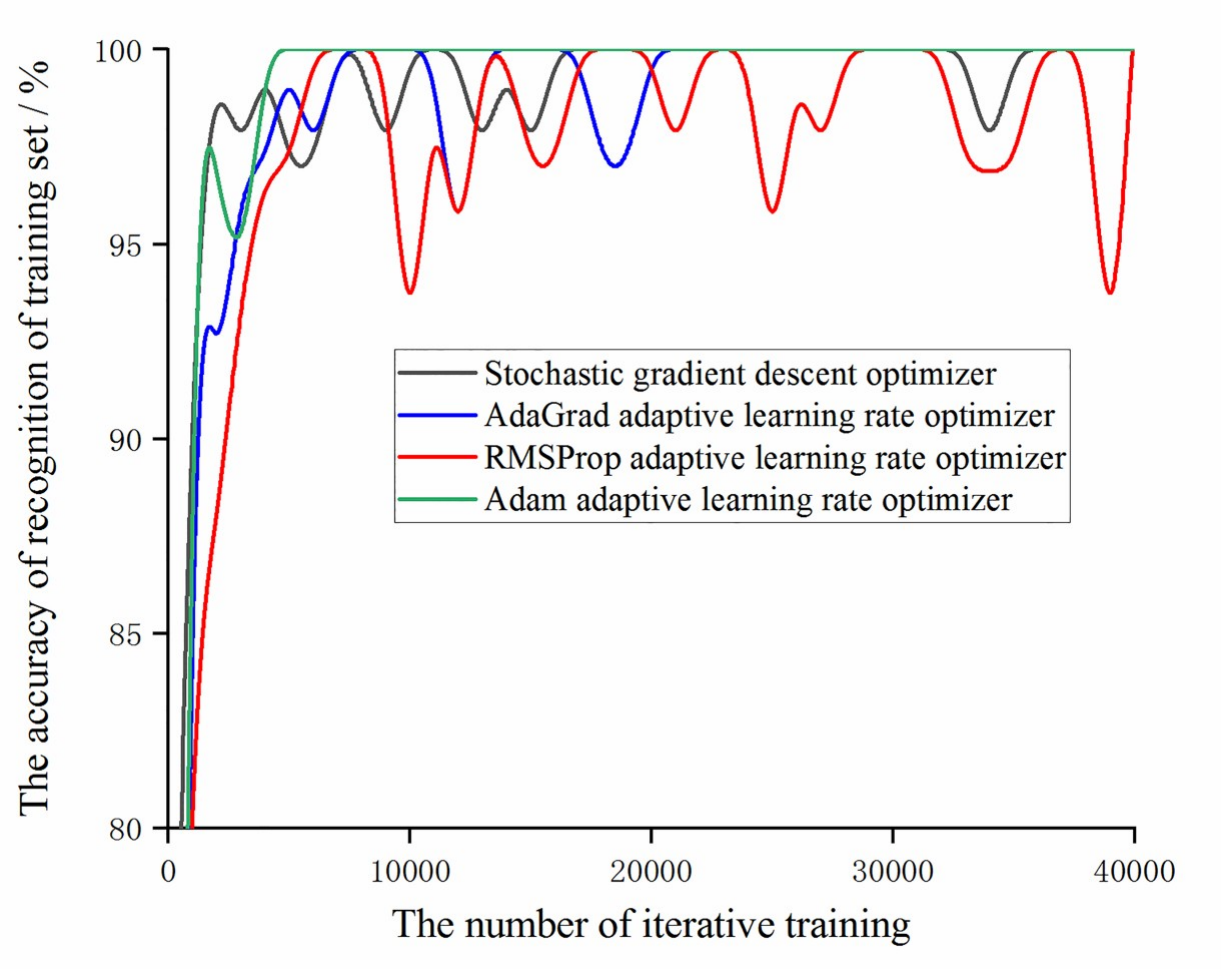
The trend of the accuracy of the training set and validation set with the number of iterative training under different optimizers.

**Fig 14:**
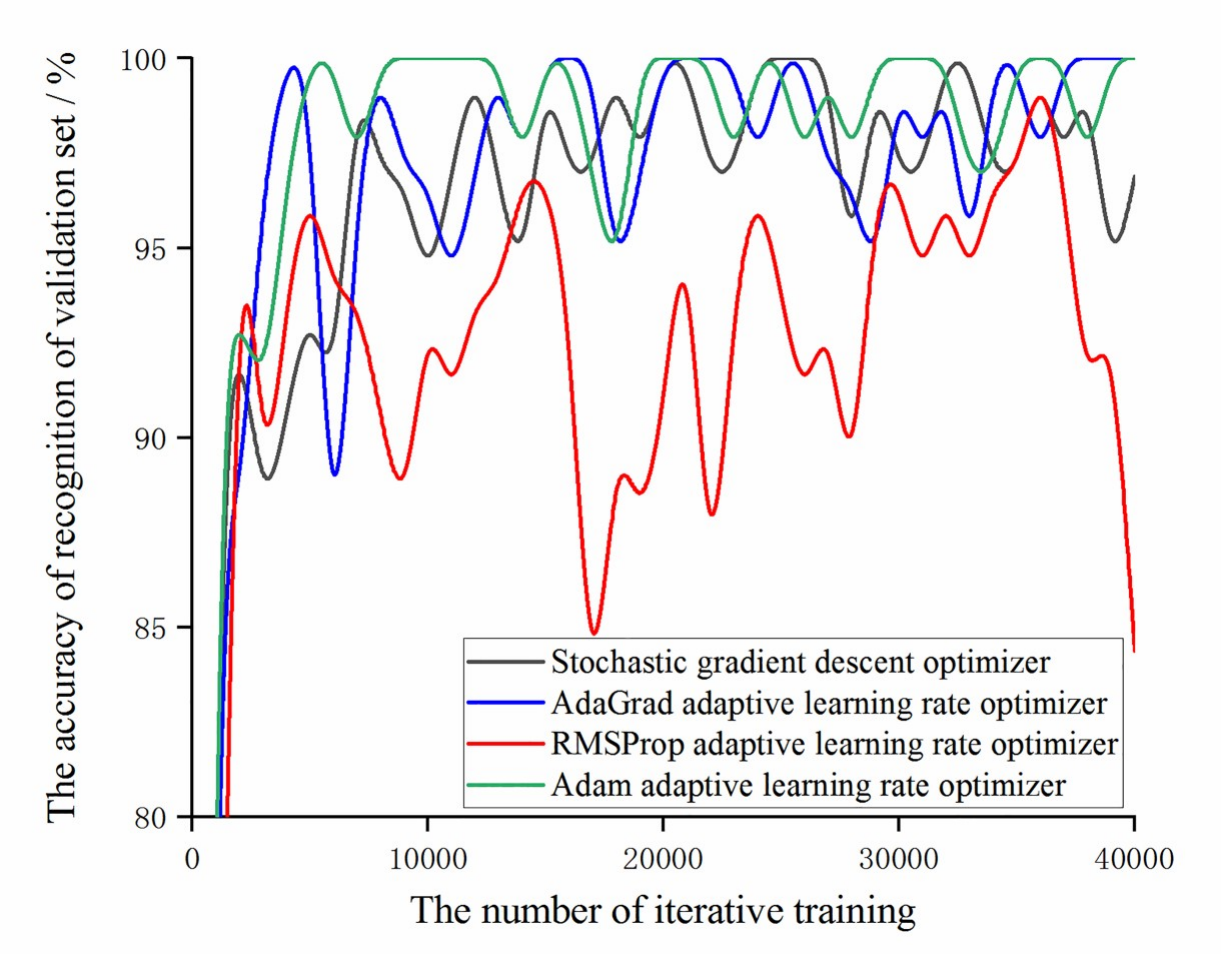
The trend of the accuracy of the validation set with the number of iterative training under different optimizers.

In order to further optimize the model and improve the accuracy, for the augmented data, the training results of the model are observed under the condition that the learning rate is set to 0.01 and a stochastic gradient descent optimizer and adaptive learning rate optimizers [33–35] based on AdaGrad algorithm, RMSProp algorithm, and Adam algorithm are used. Among them, for the adaptive learning rate optimizer based on the AdaGrad algorithm, initial_accumulator_value is set to 0.1. For the adaptive learning rate optimizer based on RMSPop algorithm, decay is set to 0.9, momentum is set to 0.0 and epsilon is set to 1e-10 by default. For Adaptive learning rate optimizer based on Adam algorithm, beta1 is set to 0.9, beta2 is set to 0.999 and epsilon is set to 1e-10 by default. In order to facilitate the comparison of the trends in the accuracy of the training set and the accuracy of the validation set under different optimizers, the result of 40,000 iterative training is taken every 1000 times to map, as shown in Figs 13 and 14.

**Fig 15:**
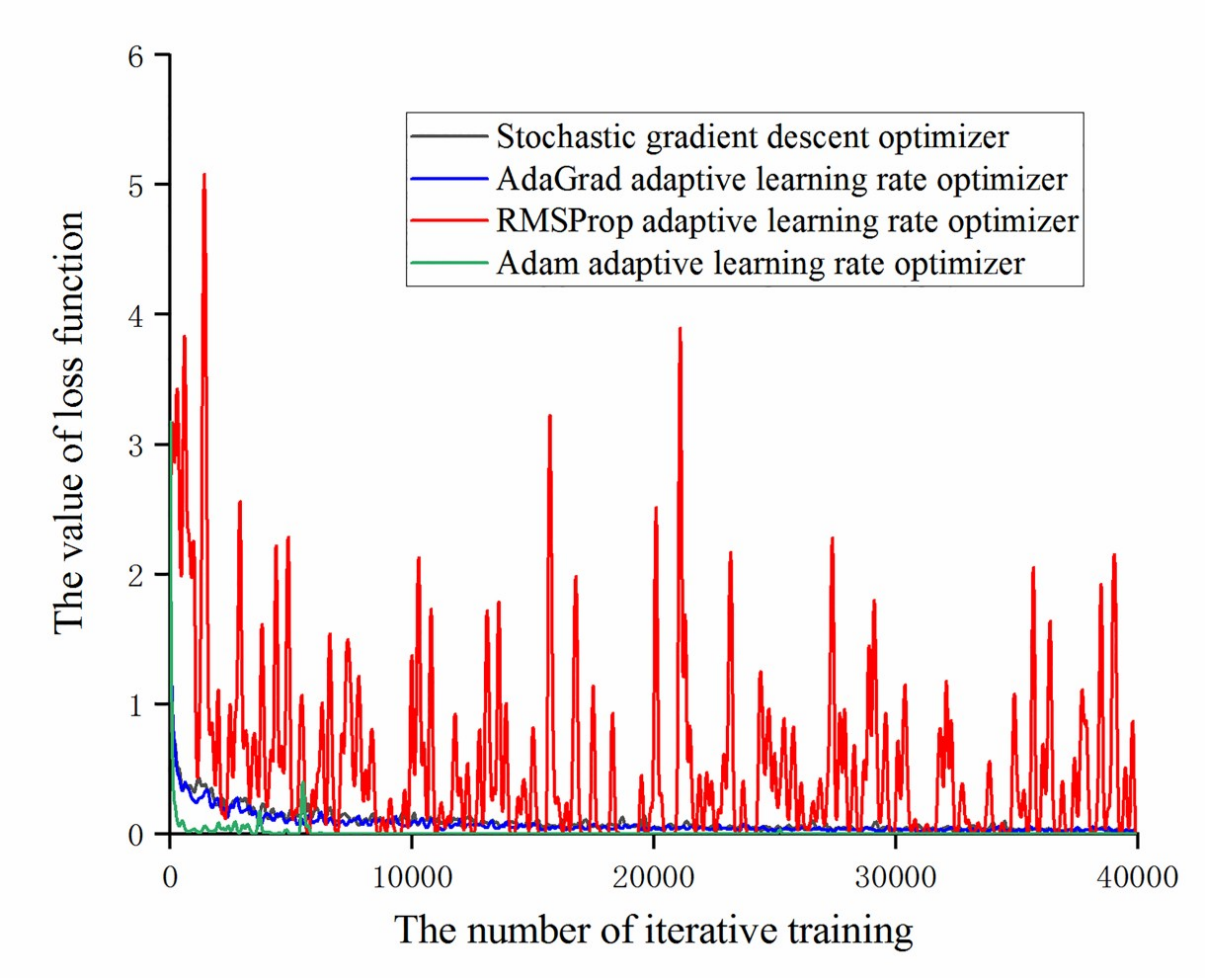
The trend of loss function value with the number of iterative training under different optimizers.

It can be obtained from Figs 13 and 14 that under the experimental samples, the accuracy with the stochastic gradient descent optimizer and the AdaGrad adaptive learning rate optimizer has almost no difference and the accuracy with the Adam adaptive learning rate optimizer is higher than the other three optimizers. At the same time, it was found that the model with the adaptive learning rate optimizer based on RMSProp algorithm has lower accuracy of recognition of training set and validation set than the other three optimizer models and the fluctuation range is large. Combining the change of the loss function value under different optimizers (as shown in Fig 15), during the training of the optimizer of the RMSProp algorithm, the value of the loss function continuously oscillates and does not converge. It can be obtained that the adaptive learning rate optimizer based on RMSProp algorithm is not applicable to this experimental sample.

**Fig 16:**
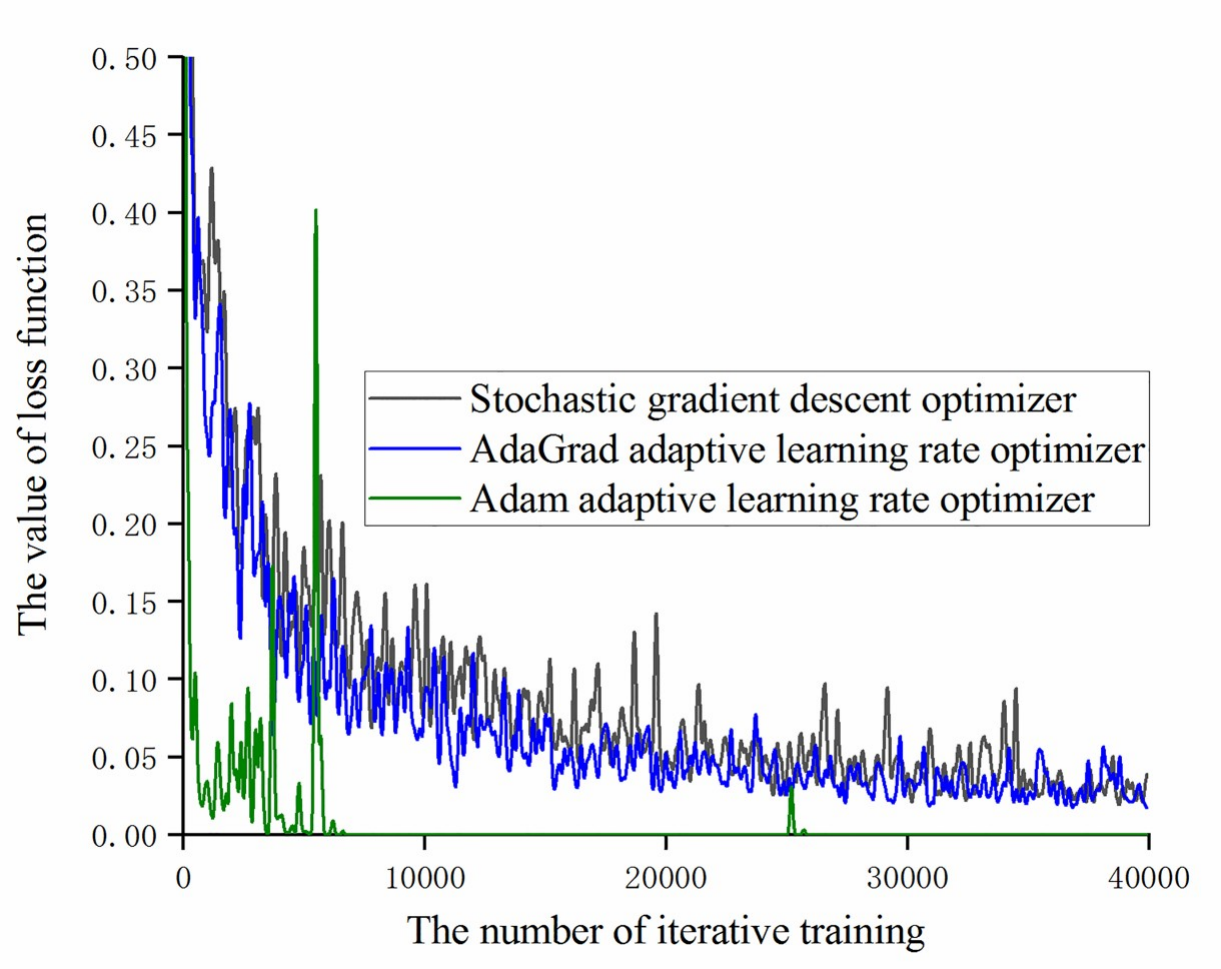
The trend of loss function value with the number of iterative training under different optimizers.

On the basis of Fig 15, remove the RMSprop adaptive learning rate optimizer and compare the loss function values of the other three optimizers, as shown in Fig 16. It can be found that the Adam adaptive learning rate optimizer has a small loss function value and fast convergence. After 7000 iterations of training, it has approached 0 and the change value is less than 0.005. The optimizer experienced a brief fluctuation during 6000 iterations, but the fluctuation range was less than 0.4, which did not affect the parameter update.

After 40,000 iterations of training were completed, the test set was used to test the models under the four optimizers and the training effect of training data set and validation data set is summarized as shown in Table 4.

**Table 4:** Comparison of model accuracy rates under different gradient descent optimizers.

Different optimizers	Training set accuracy / %	Validation set accuracy / %	Test set accuracy / %
Stochastic gradient descent optimizer	100.00	96.88	97.92
AdaGrad adaptive learning rate optimizer	100.00	100.00	98.20
RMSProp adaptive learning rate optimizer	100.00	84.38	89.25
Adam adaptive learning rate optimizer	100.00	100.00	99.04

It can be obtained from Table 4 that, for the experimental sample, the accuracy of the recognition of training set, validation set and test set of the model based on Adam adaptive learning rate optimizer are higher than the other three optimizers and the model training effect is better.

#### 3.3.4 Further optimization of results

Since the above experiment is based on 80% of dataset used for training, under normal circumstances it is easy to lead to overfitting. Therefore, the samples are divided into different groups for model training. The final accuracy rates of training set, validation set and test set are shown in Table 5.

**Table 5:** Accuracy of results of different groups.

Different ratio	Training set accuracy / %	Validation set accuracy / %	Test set accuracy / %
6: 2: 2	100.00	100.00	96.35
7: 1.5: 1.5	100.00	100.00	97.91
7.5: 1.25: 1.25	100.00	100.00	97.46
8:1:1	100.00	100.00	99.04

It can be seen from the results that when the ratio is 8:1:1, no overfitting occurs, and when the ratio of the training set is reduced, the accuracy of the test set obtained decreases. This paper analyzes this phenomenon and the main reasons are as follows.

1. Compared with big data, the number of the data set of this article is relatively small. If the proportion of the training set is further reduced, the training sample will be too small and the model fitting effect will be poor.
2. The InceptionNet-V3 used in this article has been trained by ImageNet and has good feature extraction capabilities. Unlike ordinary deep learning, transfer learning solves research problems with small samples.
3. It can be seen from the above training results that the accuracy of the test set has reached 99.04%, so it is judged that there is no overfitting.

Since the model will be applied to large sample data in the future, it is necessary to further optimize the model to achieve higher accuracy. This paper draws on the ideas and algorithms of literature [36], and considers the method of k-fold cross-validation to improve the model. Take 10% of the original data set as the final test set, and perform 10-fold cross-validation on the remaining 90% of the data. The accuracy rates of the 10 validation sets and test set obtained are shown in Table 6.

**Table 6:** 10-fold cross-validation training results.

Data set		Accuracy / %
10-fold cross-validation	Fold-1	100.00%
	Fold-2	100.00%
	Fold-3	100.00%
	Fold-4	96.88%
	Fold-5	100.00%
	Fold-6	100.00%
	Fold-7	100.00%
	Fold-8	100.00%
	Fold-9	100.00%
	Fold-10	100.00%
Average accuracy of 10-fold cross-validation validation set		99.69%
Final test set accuracy		99.74%

It can be seen from Table 6 that the model has been further optimized by using 10-fold cross-validation. Except for one validation set with an accuracy rate of 96.88%, the accuracy rates of the remaining 9 validation sets are all 100%, and the accuracy rate of the final test set reaches 99.74%, which is 0.7% higher than the optimal result in Table 5. This proves the effectiveness and superiority of the 10-fold cross-validation method.

### 3.4 Comparison of classification results of different classifiers

In order to prove the superiority of the method adopted in this paper, after the feature extraction of the image, different classifiers are used for training and the accuracy of the obtained results is shown in Table 7. The parameter settings of the classifiers here are general values or default values, and the ratio of the training set to the test set of the classifier is 4:1. The k-fold cross-validation method is not used here, because it can be seen from the results that the accuracy rate obtained is much lower than the method used in this paper, so even if other classifiers use k-fold cross-validation, they cannot achieve quite high accuracy. It can be seen that for the recognition of industrial machine parts in factories with small samples, transfer learning based on CNN has obtained very good results and can be applied in the intelligent construction of factories in the future.

**Table 7:** Accuracy of classification results of different classifiers.

Numbers	Types of classifier		Accuracy / %
1	Support vector machines (SVM)	Linear SVM	80.42
2		Quadratic SVM	82.33
3		Cubic SVM	82.43
4		Medium gaussian SVM	74.51
5		Coarse gaussian SVM	55.48
6	Nearest neighbor classifier (KNN)	Fine KNN	81.67
7		Medium KNN	70.71
8		Coarse KNN	44.74
9		Cosine KNN	79.39
10		Cubic KNN	71.26
11		Weighted KNN	74.14
12	Decision trees	Complex tree	44.27
13		Medium tree	43.54
14		Simple tree	37.33
15	Discriminant analysis	Linear discriminant	71.64
16		Quadratic discriminant	73.44
17	Ensemble	Boosted trees	57.22
18		Bagged Trees	64.41
19		Subspace discriminant	83.03
20		Subspace KNN	82.29
21		RUSBoosted Trees	61.82
22	Artificial neural networks	Back propagation neural network	88.65
23		Transfer learning based on InceptionNet-V3	99.74
24		Transfer learning based on Resnet	94.24
25		Transfer learning based on VGG16	91.50
26		Transfer learning based on Alexnet	82.14

## 4. Conclusion

Based on the transfer learning of the InceptionNet-V3 convolutional neural network model, this paper identifies and classifies 11 types of components of industrial machines. Through data augmentation, setting different learning rates and different gradient descent optimizers, the accuracy of recognition of training set accuracy, validation set and test set of the trained model are compared based on 40,000 iterations of training. In the end, after the data augmentation, the initial learning rate is taken as 0.01 and the optimizer uses the Adam adaptive learning rate gradient descent optimizer, the obtained training model is optimal. Through the analysis of the data set division ratio and 10-fold cross-validation, the final accuracy rate of the test set is 99.74%. By comparing with the accuracy of other classifiers, it can be seen that the method adopted in this paper has a better effect. This provides a basis and foundation for each factory to carry out intelligent monitoring based on its own parts and components in the future industrial background. Due to the complexity of model calculations, we will continue to study how to simplify calculations so that the model can be quickly applied in industry.

## Supporting information

S1 Appendix Program. (ZIP)

S2 Appendix Experimental data. (XLS)

S3 Appendix Description of the dataset. (DOC)
