# Supplementary material for: Recognition of industrial machine parts based on transfer learning with convolutional neural network
Source: PLoS One. 2021 Jan 28;16(1):e0245735. doi: 10.1371/journal.pone.0245735 (PMC7842930; doi:10.1371/journal.pone.0245735)
Supplement: S3 Appendix — (DOC) [file pone.0245735.s003.doc]

Description

The original image data set used in the experiment has been saved to the public database, and the access link is "https://share.weiyun.com/CTBAFgtB", which is about 7.8GB.

If you have any questions, please contact Qiaoyang Li.

E-mail：liqiaoyang2019@163.com.

Qiaoyang Li
