# Supplementary material for: Recognition of industrial machine parts based on transfer learning with convolutional neural network
Source: PLoS One. 2021 Jan 28;16(1):e0245735. doi: 10.1371/journal.pone.0245735 (PMC7842930; doi:10.1371/journal.pone.0245735)
Supplement: S1 Appendix — (ZIP) [file pone.0245735.s001.zip › S1 Appendix/sotfware and hardware environment.doc]

The experiments were completed under the software environment of Python 3.7.0 and TensorFlow 1.15.0. The hardware environment is that the CPU uses Intel Corei5-6200U and the main frequency is 2.3GHz; the GPU uses NVIDIA GeForce 950M with 2GB video memory.
